# Ameloblastic carcinoma: An analysis of 12 cases with a review of the literature

**DOI:** 10.3892/ol.2014.2230

**Published:** 2014-06-05

**Authors:** JIA LI, HONGMING DU, PENG LI, JINGKUI ZHANG, WEIDONG TIAN, WEI TANG

**Affiliations:** 1 State Key Laboratory of Oral Diseases, West China College of Stomatology, Sichuan University, Chengdu, Sichuan 610041, P.R. China; 2 Department of Oral and Maxillofacial Surgery, West China College of Stomatology, Sichuan University, Chengdu, Sichuan 610041, P.R. China

**Keywords:** ameloblastic carcinoma, diagnosis, treatment, radiotherapy

## Abstract

The diagnosis of ameloblastic carcinoma is often difficult and the optimal treatment methods remain controversial. The current study retrospectively investigated the optimal diagnosis and treatment methods of 12 ameloblastic carcinoma patients at the West China Hospital of Stomatology, Sichuan University (Chengdu, China), and 20 patients selected from the PubMed database, were reviewed. The clinical features, diagnosis and outcome of the different treatments were evaluated. Ameloblastic carcinoma occurred in 12 out of a total of 538 ameloblastoma patients; the majority were of the primary type. Of the 538 ameloblastoma patients, 294 were male, 244 were female with a male to female ratio of 1.2:1. The predilection age is 20–30 years, which accounts for 40% of the total. In total, 461 cases were in the mandible and 77 were located in the maxilla. The cure rate of the primary type and the recurrence rate of the secondary type tumors were higher in the patients from the West China Hospital of Stomatology compared with those reported in the literature. In particular, a case with a long-term survival of 30 years is presented, which is considered to be relatively rare. The evolution of the clinical course has experienced three stages: Ameloblastoma (1978) followed by metastatic ameloblastoma (2000) and finally ameloblastic carcinoma (2008). To avoid recurrence, wide local excision with postoperative radiation therapy is required. While novel therapeutic regimens should also be considered as appropriate, including carbon ion therapy and Gamma Knife stereotactic radiosurgery. However, controlled studies with larger groups of patients are required to increase the accuracy of results.

## Introduction

Ameloblastic carcinoma is a relatively rare type of tumor. According to the World Health Organization (WHO), the carcinoma can be classified as metastasizing (malignant) ameloblastoma or ameloblastic carcinoma (1). Metastasizing ameloblastoma is defined as an ameloblastoma, which metastasizes and the primary and metastatic tissues demonstrate benign histological features (2). Whereas ameloblastic carcinoma exhibit malignant features, such as cellular atypia and mitosis (3). Ameloblastic carcinoma consists of two subtypes; primary and secondary. The primary type demonstrates malignancy in the primary tumor with characteristics of ameloblastoma and cytologic atypia. The secondary type consists of malignant changes, which originate in a previously existing ameloblastoma, regardless of the presence or absence of metastasis. The secondary type of ameloblastic carcinoma can be divided into two further subtypes. The intraosseous type arises within a pre-existing benign intraosseous ameloblastoma and the peripheral type arises within a pre-existing benign peripheral ameloblastoma (4). Angiero *et al* (5) reported that ameloblastic carcinoma possess unique histopathological features. At the early stages of malignancy or dedifferentiation, epithelial tumor nests and islands surrounded by a layer of stellate basaloid cells are observed in the mesenchymal tissue. Mubeen *et al* (6) recognized that these cells exhibit malignant features, such as cellular pleomorphism, mitoses, focal necrosis, perineural invasion and nuclear hyperchromatism. Furthermore, ameloblastic carcinoma exhibit histological features of ameloblastoma and carcinoma.

Ameloblastic carcinoma is an uncommon tumor type, and therefore, the clinical characteristics, appropriate treatment and response rates have not been well characterized. Benlyazid *et al* (7) retrospectively reviewed 66 patients with ameloblastic carcinoma that were reported between 1927 and 2006, and the majority exhibited lung metastasis, which indicated the requirement for systemic therapy. As a relatively rare malignant tumor, further detailed and systematic studies regarding ameloblastic carcinoma are required.

## Patients and methods

### Patients

In total, 12 patients with ameloblastic carcinoma who were treated at the West China Hospital of Stomatology, Sichuan University (Chengdu, China) between 2000 and 2008 (Table I), and 20 more cases reported between 2005 and 2010 identified by searching PubMed (http://www.ncbi.nlm.nih.gov/pubmed; Table II) were retrospectively reviewed.

### Classification of lesions

The ameloblastic carcinomas were classified as primary or secondary by a reviewing pathologist using the 2005 WHO criteria (8). The gender, age, primary site, surgical procedures, pathology and outcome were identified. Primary and metastatic lesions were confirmed by X-ray, computed tomography (CT) and pathological examination. Written informed consent was obtained from all patients and approval of the study was obtained from the Ethics Committee of Sichuan University. The study was also approved by the West China Hospital of Stomatology Review and Ethics Board.

### Procedures

An extended jaw resection was performed if the X-ray and CT scan demonstrated destruction of the cortical bone, involvement of the periosteum, or invasion of soft tissue. In cases where the tumor invaded the cortical bone and exhibited no invasion of the soft tissue, a partial jaw resection was performed. A marginal ostectomy was performed for tumors that were limited to the alveolar bone without cortical invasion.

## Results

### Patients from the West China Hospital of Stomatology

A total of 12 patients with ameloblastic carcinoma were treated at the West China Hospital of Stomatology between 2000 and 2008. The incidence of ameloblastic carcinoma was relatively uncommon compared with that of ameloblastoma (12/538; 2.23% of overall cases). The male:female ratio was 5:1 and the mean age was 44 years (range, 30–75 years). Tumors occurred more frequently in the mandible than in the maxillary (11:1) and eight tumors were primary and four were secondary.

All of the patients with primary tumors (8/8) were cured following extended resection (2), partial resection (5), or marginal ostectomy (1), while only one patient with a secondary tumor was cured (1/4; 25%). Patients exhibiting secondary tumors were treated by extended (2) or partial resection (2); two of the four (50%) patients developed a local recurrence and one (1/4; 25%) exhibited metastases. No patients underwent cervical lymph node dissection, chemotherapy or radiotherapy. The cure rate of males and females was 80% (8/10) and 100% (2/2), respectively. The cure rate was 75% (3/4) following extended jaw resection, 71% (5/7) following partial jaw resection and 100% (1/1) following marginal ostectomy.

**Figure 1:**
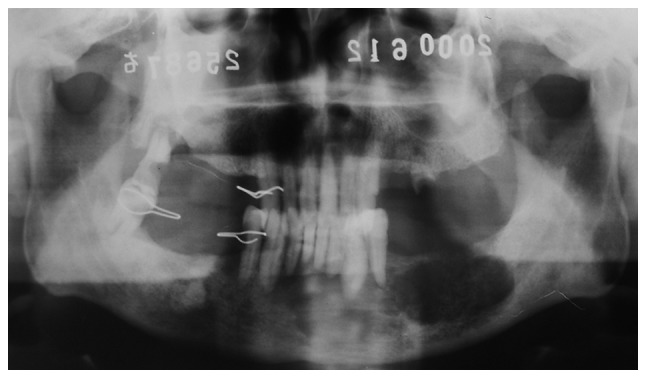
Preoperative panoramic view of an ameloblastic carcinoma patient showing a low-density signal from C3 to the leading edge of the left mandibular ramus.

**Figure 2:**
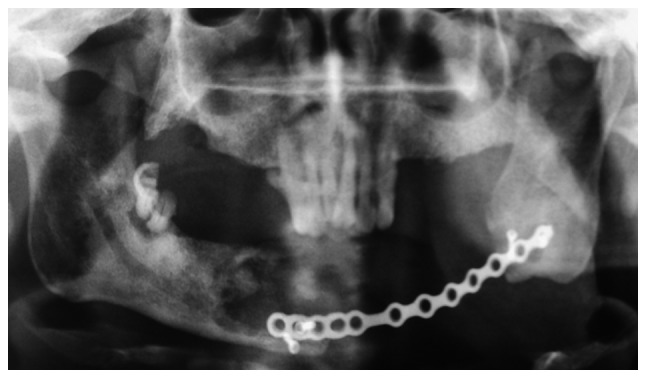
Postoperative panoramic view of one patient showing a large area of bone loss in the mandible. A relatively low-density signal was observed in the C3–C6 area. The reconstruction plate is shown in place, with three loosening screws. Additionally, potential tumor involvement of the surgical margins was observed.

**Figure 3:**
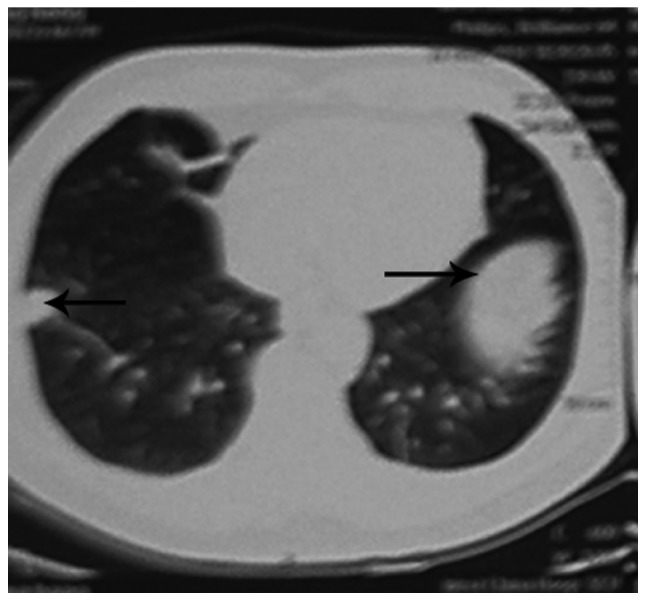
Chest radiograph of one patient showing metastases in the bilateral lower lobes of the lung (black arrows).

**Figure 4:**
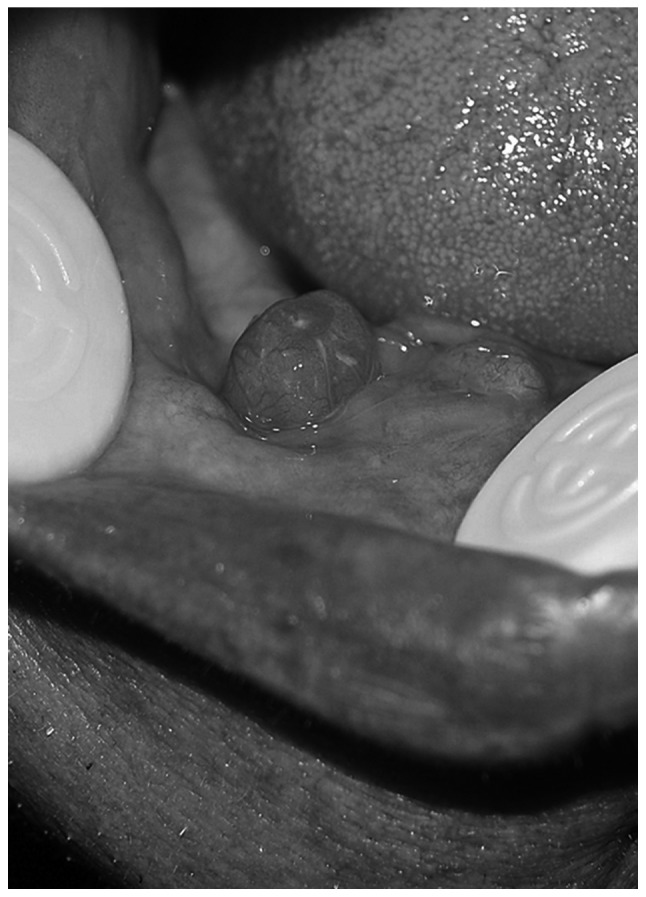
Clinical image of one patient demonstrating an exophytic mass on the gum of the right mandible around the C4.

**Figure 5:**
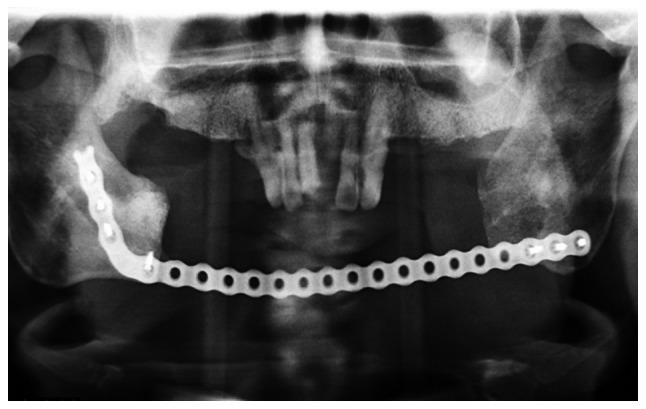
Postoperative panoramic image demonstrating bone loss between the bilateral mandibular ramus and the reconstruction plate was placed in the defect area to aid with repair.

A 75-year-old male was diagnosed with left jaw ameloblastoma. Following curettage in 1978 at the West China Hospital of Stomatology, the chest radiograph was normal. However, 22 years later, panoramic radiographs demonstrated bone destruction (Fig. 1) and a chest X-ray demonstrated a solitary nodule. A needle biopsy of the lung nodule revealed ameloblastoma and the patient underwent a partial mandibulectomy; the pathological examination demonstrated metastatic ameloblastoma. Eight years later, the tumor recurred (Fig. 2) and a chest CT showed bilateral lung nodules (Fig. 3). Pathological review of the needle biopsy demonstrated ameloblastic carcinoma. Subsequently, the patient underwent an extended mandible resection of the ameloblastic carcinoma (secondary type; Figs. 4 and 5). The patient experienced various stages of disease development: a malignant transformation from ameloblastoma, metastatic ameloblastoma, to ameloblastic carcinoma, and survival with a tumor for 30-years, which is particularly rare.

### Review of cases from the literature

In total, 20 patients with ameloblastic carcinoma, which were reported between 2005 and 2010 were identified by searching PubMed (Table II). The postoperative follow-up ranged between six and 48 months. The male:female ratio was 4:1 and the mean patient age was 56.3 years (range, 10–91 years). A total of 10 patients exhibited maxillary tumors and 10 had mandibular tumors. Furthermore, 17 patients had primary tumors and three exhibited secondary tumors. Among the patients with primary tumors, 2/17 exhibited cervical lymph node metastases and 1/17 exhibited lung metastases. Among the patients with secondary tumors, 2/3 exhibited lymph node metastases.

The cure rate of males and females was 87.5% (14/16) and 100% (4/4), respectively. The cure rate was 88.2% (15/17) in patients with primary tumors and 100% (3/3) in patients with secondary tumors (3/3). In addition, the cure rate was 100% (15/15) following extended jaw resection and 60% (3/5) following partial jaw resection. Two patients with lymph node metastasis were also treated with radical neck dissection and seven patients with lymph node metastasis underwent prophylactic lymph node dissection. The cure rate was 88.9% (8/9) following lymph node dissection and 90.9% (10/11) without lymph node dissection. Chemotherapy and radiotherapy were administered for lung metastases, and radiotherapy alone was administered for lymph node metastasis. The response rates were 100% (1/1) for chemotherapy and radiotherapy, 100% (4/4) for radiotherapy, and 86.7% (13/15) without chemotherapy or radiotherapy (Table III). For the 17 patients with primary tumors, the cure rate was 100% (13/13) following extended resection and 50% (2/4) following partial resection (2/4); one patient succumbed to the disease and one developed metastases. The cure rate was 85.7% (6/7) following lymph node dissection and 90% (9/10) without lymph node dissection. Only primary tumors were treated with radiotherapy and chemotherapy. The cure rate was 100% (5/5) for patients treated with chemotherapy and radiotherapy, and 83.3% (10/12) for patients treated with surgery alone. The cure rate for secondary tumors was 100% (3/3); two patients were treated with an extended resection and one was treated with a partial resection. The cure rate was 100% (2/2) for patients with secondary tumors following lymph node dissection, and 100% without lymph node dissection (1/1).

## Discussion

The evolution of ameloblastoma to ameloblastic carcinoma is controversial (14). The exact mechanism of the malignant transformation is currently unknown due to the limited number of reports. One study has shown that malignant transformation requires a relatively long duration, and multistage carcinogenesis is a reasonable suggestion as to the underlying mechanism of malignant transformation (20). Makiguchi *et al* (21) demonstrated that the malignant and benign regions are distinguishable by preoperative 18F-α-methyl tyrosine positron emission tomography and magnetic resonance imaging to avoid excessive resection, severe functional loss and a poor facial appearance. However, the exact mechanism requires further investigation. The present study presents the progression from benign ameloblastoma to metastatic ameloblastoma and eventually to ameloblastic carcinoma in a 75-year-old male. Surgical treatment alone was effective for this patient. Rare diseases, including the ameloblastoma to ameloblastic carcinoma spectrum, require randomized multicenter studies in order to define novel and improved treatments.

The most common clinical manifestation of ameloblastoma is a swollen, occasionally painful, jaw. However, certain tumors grow rapidly and limit the opening of the mouth. In addition, rapid tumor growth may perforate the cortical bone and extend into the soft tissue, causing pain and paresthesia. Akrish *et al* (18) retrospectively analyzed 37 patients with ameloblastic carcinoma, which were reported between 1984 and 2007. In these patients, the male:female ratio was 2:1, the mean age was 52 years (range, 15–84 years), and the maxillary:mandible tumor ratio was 13:25. In the current study PubMed was used and 20 cases of ameloblastic carcinoma (reported between 2005 and 2010) and staged according to the 2005 WHO classification were identified. The male:female ratio was 4:1, maxillary:mandible tumor ratio was 10:10, and mean age was 56.3 years (range, 10–91 years). A total of 17 patients had primary tumors and three exhibited secondary tumors. By contrast, the majority of the patients treated at the West China Hospital of Stomatology were male and aged between 30 and 45 years. In addition, the majority of tumors were in the mandible (92%), including eight primary and four secondary tumors.

Ameloblastic carcinoma occurs in a wide range of age groups with no apparent gender predilection. The most commonly involved area is the mandible and the most common pathology is the primary type. All the secondary tumors presented in the present study and in the identified literature were of the intraosseous type. The peripheral type of ameloblastic carcinoma, arising within a pre-existing benign peripheral ameloblastoma, was relatively rare.

The optimal treatment for ameloblastic carcinoma remains unknown. In the present study, the cure rate of the primary tumors was higher than that observed in the literature (100 vs. 88.2%), however, the recurrence rates of the secondary tumors were higher than those presented in the literature (16.7 vs. 0%). The incidence of lymph node metastases in the present study was less frequent than in the literature (0 vs. 10%). Two patients with lung metastases were identified in the present study group (primary type) and the literature (secondary type). Ameloblastic carcinoma exhibits the histological features and behavior of malignancy, and therefore, definitive surgical treatment is required (20). In the literature the cure rate following extended jaw resection was 100% for primary and secondary tumors. In the present group of patients, the recurrence rate following partial resection was 50%. Therefore, these results support the use of extended jaw resection to prevent local recurrence. Yoon *et al* (14) reported a recurrence rate of 92.3% following curettage alone, and 28.3% following partial resection. Extended jaw resection involves a margin of resection of 2–3 cm, including the normal bone, periosteum and soft tissue. This approach has been found to reduce the local recurrence rate by 15% when compared with partial resection (22). Naik and Kale (17) also reported that surgical stimulation and incomplete resection may induce the degeneration of early lesions, and explain the higher recurrence rate following partial resection, which was identified in patients with secondary ameloblastic carcinoma compared with primary.

At present, the use of cervical lymph node dissection with ameloblastic carcinoma is under review. Jeremic *et al* (12) proposed the use of parotid gland resection and regional lymph node dissection to achieve adequate margins. Angiero *et al* (5) argued that since metastases is able to occur via the blood stream, cervical lymph node dissection should not be routinely performed. In the present study, patients with primary tumors and no prophylactic lymph node dissection exhibited higher cure rates. In addition, the cure rate of secondary tumors was higher compared with the cases in the literature. However, due to the small number of patients, elective neck dissection is not recommended for this type of lesion. Yoon *et al* (14) also revealed that neck dissections should not be recommended for primary or secondary lesions without evidence of cervical lymph node involvement.

The literature review identified four patients that were treated with radiotherapy and one, which was treated with a combination of chemotherapy and radiotherapy. All five patients had primary tumors. The cure rate was 100% for the combination of chemotherapy and radiotherapy, and 100% for radiotherapy alone. Chemotherapy has not been indicated as a primary treatment (16). Patients with secondary tumors exhibit a higher rate of recurrence and metastasis and to date, chemotherapy has shown no favorable results for local control (23). In addition, few chemotherapy reports are available and its role is yet to be confirmed (24). Furthermore, the combination of chemotherapy and radiotherapy to treat patients with secondary tumors requires further evaluation.

It is generally acknowledged that ameloblastic carcinoma is considered to be a radioresistant tumor. However, pre- or postoperative radiotherapy may reduce the size of ameloblastic carcinomas (17). Dhir *et al* (25) reported that 50% of postoperative patients developed local recurrence or metastasis, and could be treated with radiotherapy. Radiotherapy alone is appropriate for patients who are not surgical candidates, or exhibit advanced local or metastatic disease.

Certain novel treatment methods have gradually been put forward. Ion beam therapy, which delivers high doses to the target tumor while sparing the normal surrounding tissues, is a novel therapy for cancer. Recently, Jensen *et al* (26) reported the use of carbon ion therapy for recurrent ameloblastic carcinoma over four weeks, which showed no recurrence following three months. In addition, compared with the conventional radiotherapy, carbon ion therapy exhibits less severe complications. Perera *et al* (23) considered Gamma Knife stereotactic radiosurgery as a promising option for instances where tumors present in surgically complex regions.

In conclusion, ameloblastic carcinoma is a relatively rare type of tumor, occurring in only 2.23% (12/538) of patients presenting with ameloblastoma at the West China Hospital of Stomatology. Ameloblastic carcinoma exhibit an aggressive clinical behavior, including rapid tumor growth, painful swelling and perforation of the cortex. The proposed mechanisms underlying the transformation of a classic benign ameloblastoma into a malignant tumor remain controversial. It has been indicated that wide local excision with postoperative radiation therapy should be employed. However, novel therapeutic regimens must be considered, including carbon ion therapy and Gamma Knife stereotactic radiosurgery. Controlled studies with larger groups of patients are required to increase the accuracy of results.

## Figures and Tables

**Table I:** Review of 12 cases of ameloblastic carcinoma with a follow-up of >36 months from the West China Hospital of Stomatology between 2000 and 2008.

Case, n	Gender/age, years	Type	Location	Therapy	Met.	Re.	Follow-up, months	Prognosis
1	M/36	S	Mandible	Partial resection	-	Y	120	Re.
2	F/40	S	Mandible	Expand resection	-	-	120	DF
3	M/47	S	Maxillary	Partial resection	-	Y	108	Re.
4	M/61	P	Mandible	Partial resection	-	-	108	DF
5	M/40	P	Mandible	Expand resection	-	-	96	DF
6	F/39	P	Mandible	Partial resection	-	-	84	DF
7	M/42	P	Mandible	Expand resection	-	-	72	DF
8	M/46	P	Mandible	Partial resection	-	-	60	DF
9	M/32	P	Mandible	Partial resection	-	-	60	DF
10	M/30	P	Mandible	Marginal ostectomy	-	-	48	DF
11	M/35	P	Mandible	Partial resection	-	-	36	DF
12	M/75	S	Mandible	Expand resection	Lung	-	36	Met.

Met., metastasis; Re., recurrence; S, secondary type; P, pimary type; Y, yes; DF, disease free.

**Table II:** Review of 20 cases of ameloblastic carcinoma from an evidence-based literature review between 2005 and 2010.

Case, n	First author (year) [ref]	Gender/ age, years	Type	Location	Therapy	Met.	Re.	Follow-up, months	Outcome
1	Lucca *et al* (2010) [9]	M/69	P	Maxillary	Jaw extended resection	-	-	11	DF
2	Karakida *et al* (2010) [10]	M/43	S	Mandible	Jaw extended resection, neck dissection	-	-	46	DF
3	Jindal *et al* (2010) [11]	M/60	S	Mandible	Jaw extended resection	-	-	19	DF
4	Jeremic *et al* (2010) [12]	M/58	P	Mandible	Jaw extended resection, neck dissection, radiation and chemotherapy following 9 months	Lung	-	21	DF
5	Devenney-Cakir *et al* (2010) [13]	M/16	P	Mandible	Partial resection, lymph node dissection	-	-	48	Met. (lung)
6	Yoon *et al* (2009) [14]	M/63	P	Maxillary	Jaw extended resection, radiation	-	Y	13	DF
		F/73	P	Maxillary	Jaw extended resection	-	-	31	DF
		M/61	P	Maxillary	Jaw extended resection	-	-	13	DF
		M/46	P	Mandible	Jaw extended resection, neck dissection, radiation	LN	Y	18	DF
		M/58	P	Maxillary	Jaw extended resection, neck dissection	-	-	12	DF
		M/65	P	Mandible	Jaw extended resection, neck dissection, radiation	LN	-	13	DF
7	Ismail *et al* (2009) [15]	F/21	P	Mandible	Jaw extended resection, neck dissection	-	-	36	DF
8	Yazici *et al* (2008) [16]	M/10	P	Maxillary	Jaw extended resection, radiation	-	-	6	DF
9	Angiero *et al* (2008) [5]	M/68	P	Maxillary	Jaw extended resection	-	-	6	DF
10	Ward *et al* (2007) [4]	M/64	P	Maxillary	Jaw extended resection	-	-	42	DF
11	Naik and Kale (2007) [17]	M/70	P	Maxillary	Partial resection	-	-	12	DF
12	Benlyazid *et al* (2007) [7]	M/90	P	Maxillary	Partial resection	-	-	25	STD
13	Akrish *et al* (2007) [18]	M/80	S	Mandible	Partial resection, neck dissection	-	-	12	DF
14	Suomalainen *et al* (2006) [2]	F/21	P	Mandible	Partial resection, neck dissection	-	-	30	DF
15	Miyake *et al* (2006) [19]	F/91	P	Mandible	Jaw extended resection	-	-	6	DF

Met., metastasis; Re., recurrence; P, primary type; S, secondary type; LN, lymph node; Y, yes; DF, disease free; STD, succumbed to disease.

**Table III:** Ameloblastic carcinoma cure rates at the West China Hospital of Stomatology and from the literature.

	Cure rate, n (%)	
	
Variable	West China Hospital (n=12)	PubMed literature (n=20)
Gender		
Male	10 (80.0)	16 (87.5)
Female	2 (100.0)	4 (100.0)
Type		
Primary	8 (100.0)	17 (88.2)
Secondary	4 (25.0)	3 (100.0)
Therapy		
Extended resection	4 (75.0)	15 (100.0)
Partial resection	7 (71.4)	5 (60.0)
Marginal ostectomy	1 (100.0)	N/A
Neck dissection	N/A	9 (88.9)
Primary type	N/A	7 (85.7)
Secondary type	N/A	2 (100.0)
No neck dissection	N/A	11 (90.9)
Primary type	N/A	10 (90.0)
Secondary type	N/A	1 (100.0)
Radiation and chemotherapy	N/A	5 (100.0)
Primary type	N/A	5 (100.0)
Secondary type	N/A	N/A
No radiation and chemotherapy	N/A	15 (86.7)
Primary type	N/A	3 (83.3)
Secondary type	N/A	3 (100.0)

N/A, not applicable (treatment was not used in this group).
